# Role of Physiology, Immunity, Microbiota, and Infectious Diseases in the Gut Health of Poultry

**DOI:** 10.3390/vaccines10020172

**Published:** 2022-01-22

**Authors:** Samiru S. Wickramasuriya, Inkyung Park, Kyungwoo Lee, Youngsub Lee, Woo H. Kim, Hyoyoun Nam, Hyun S. Lillehoj

**Affiliations:** 1 Animal Bioscience and Biotechnology Laboratory, Beltsville Agricultural Research Center, Agricultural Research Service, United States Department of Agriculture, Beltsville, MD 20705, USA; samiru.sudharaka@usda.gov (S.S.W.); inkyung.park@usda.gov (I.P.); kyungwoolee@konkuk.ac.kr (K.L.); youngsub.lee@usda.gov (Y.L.); woohyun.kim@gnu.ac.kr (W.H.K.); hyoyoun.nam@usda.gov (H.N.); 2 Department of Animal Science and Technology, Konkuk University, 120 Neungdong-ro, Gwangjin-gu, Seoul 05029, Korea; 3 College of Veterinary Medicine and Institute of Animal Medicine, Gyeongsang National University, Jinju 52828, Korea

**Keywords:** chicken, gut health, gut diseases, gut–brain axis, gut integrity, immunity, microbiota, oxidative stress, alternatives to antibiotics

## Abstract

“Gut health” refers to the physical state and physiological function of the gastrointestinal tract and in the livestock system; this topic is often focused on the complex interacting components of the intestinal system that influence animal growth performance and host-microbial homeostasis. Regardless, there is an increasing need to better understand the complexity of the intestinal system and the various factors that influence gut health, since the intestine is the largest immune and neuroendocrine organ that interacts with the most complex microbiome population. As we face the post-antibiotic growth promoters (AGP) era in many countries of the world, livestock need more options to deal with food security, food safety, and antibiotic resilience to maintain agricultural sustainability to feed the increasing human population. Furthermore, developing novel antibiotic alternative strategies needs a comprehensive understanding of how this complex system maintains homeostasis as we face unpredictable changes in external factors like antibiotic-resistant microbes, farming practices, climate changes, and consumers’ preferences for food. In this review, we attempt to assemble and summarize all the relevant information on chicken gut health to provide deeper insights into various aspects of gut health. Due to the broad and complex nature of the concept of “gut health”, we have highlighted the most pertinent factors related to the field performance of broiler chickens.

## 1. Introduction

With the global population anticipated to reach 10 billion by 2050, the demand for food production will increase by 70% [1]. According to the World Health Organization (WHO), the annual meat production is expected to increase by additional 200 million tons to reach the 525 million tons that are needed to feed the growing population globally. Meeting this demand will be the collective responsibility of agricultural scientists and food producers to meet the goal of producing a nutritious, safe, affordable, and sustainable food supply. Furthermore, increasing urbanization and the changing economic status of the next generation are likely to require high-quality food products that are safe and nutritious for consumption. To meet the global food demand, animal husbandry faces constant pressure to produce high-quality protein under a different set of challenges, such as animal welfare, mechanization and robotization of the industry, political instability, and environmental pollution.

In recent years, animal husbandry has improved the growth potential of animals as a result of science-based genetic selection and dietary sub-therapeutic supplementation of antibiotics growth promoters that have successfully facilitated the growth potential of agricultural animals with enhanced feed efficiencies [2,3]. However, an increase in antibiotic resistance in humans and animals has led to a regulatory restriction on the use of antibiotics in livestock production. With the imposed ban and consumer pressure on meat production without antibiotics, farmers are facing greater challenges to reach the production targets in the intense farming systems [2].

To maintain the sustainability of the intense animal production system while catering to the demand for safe and nutritious food, alternative strategies to antibiotics are emerging. Scientists have proposed some promising alternatives to animal production to mimic antibiotic growth promoters [2], and many have been successfully applied in the commercial production of animal meats. These include prebiotics, probiotics, synbiotics, organic acids, essential oils, hyper-immune IgY antibodies, enzymes, and phytogenic feed additives, which are now being used commercially. Extensive information on enhancing gut health via modulation of the gut microbiota [2,4] is becoming available, although more fundamental research is needed to transfer these basic findings to microbiota manipulations to improve animal performance at the farm level.

The importance of gut health in animals goes beyond the physical barrier of the gut [5]. Although substantial scientific findings on gut health-related topics have been published, the understanding of its composition, importance, regulation, or interactions is still at the rudimentary stage. Nonetheless, the fact that gut health has been a matter of contention in scientific research reflects its importance in various aspects of animal nutrition and production. The term “gut health” encompasses broader areas of the physical state and physiological function of the many parts of the gastrointestinal (GI) tract [6]. Bischoff [7], while proposing the definition of gut health, used the definition as outlined by the WHO and defined the “gut health in human medicine” as a state of physical and mental well-being in the absence of GI complaints that require the consultation of a doctor, in the absence of indications or risks of bowel diseases, and the absence of confirmed bowel diseases. This definition of gut health in human medicine may be applicable to animals as well, including poultry, due to the similarity in structure, physiology, and function of the gut, despite quantitative and qualitative differences that may exist between animals and humans. Kogut and Arsenault [8] defined gut health as “the absence/prevention/avoidance of disease so that the animal is able to perform its physiological functions in order to withstand exogenous and endogenous stressors”. Later, Celi et al. [9] proposed the definition of gut health as “a steady-state, where the microbiome and the intestinal tract exist in symbiotic equilibrium, and where the welfare and performance of the animal are not constrained by intestinal dysfunction”. These definitions show that gut health is a holistic term and point out the importance of complex interactions of various components of the gut interacting with the host animal and the environment to maintain homeostasis. Indeed, Jha et al. [10] revised the early definition by Conway [11] on gut health being the function of three major components (i.e., diet, the mucosa, and the commensal microbiota); they suggested that gut health is holistically the function of four major components, including diet (i.e., nutrition), mucosa, microbiome, and the immune system, in a holistic way. Thus, understanding the four major components is a prerequisite to defining gut health and will also expand our knowledge of their functions in determining gut health. Further, a wide range of intrinsic and extrinsic factors [9] affecting gut health (e.g., gut components) need to be better elucidated. Although recent attempts have made it possible to define gut health with collated scientific publications [8,9,10], a comprehensive review on gut health from the perspective of nutritionists, physiologists, immunologists, and veterinarians is not yet available. In this review, we aimed to summarize the current understanding of the structure and physiology of the gut and the intrinsic and extrinsic factors affecting gut health in chickens.

## 2. Methods

This review was performed in a descriptive and unbiased manner through an in-depth review of the scientific literature using major online databases, including PubMed, Google Scholar, Scopus, and Research Gate. The key search terms for this review based on each sub-topic and some of the major terms included: poultry, gut health, gut diseases, gut–brain axis, gut integrity, immunity, microbiota, oxidative stress, and alternatives to antibiotics.

## 3. Structure and Physiology

### 3.1. Chicken Gut and Its Relation to Gut Health

Understanding the structure and physiology of the chicken intestine is important to manage optimum gut health, since the gut has the largest surface of the body that is exposed to the external environment and is the location where dietary components and external microbes come in contact with the host [12]. Although all poultry species possess similar gut structures, the chicken gut has unique anatomical, histological, and physiological features, which are distinct from other avian species, as well as mammals. Notably, the intestinal tract in chickens is relatively shorter than that in mammalian animals, and hence, chickens have a lesser retention time of feed and digestive capacity than in their mammalian counterparts [13]. Therefore, it is important to maintain the optimal functionality of chickens with a good gut health status as they develop to maximize the feed efficiency and growth.

Similar to other avian species, chickens use their beaks to obtain feed. Compared with other poultry species, chickens have a heavily keratinized tongue, owing to the absence of a stratum granulosum [14]. Interestingly, antimicrobial peptides (AMPs), such as beta-defensins, gallinacin-3, and gallinacin-6, are expressed in chicken tongue, along with the presence of Langerhans cells, indicating their crucial role in the first line of immune defense against pathogens locally [15]. Secretions from the salivary glands contain salivary enzymes, such as amylase, and the salivary mucus initiates a chemical/mechanical defense mechanism against pathogens to maintain optimum gut health [15].

In chickens, feedstuff, once ingested through the mouth, passes into the crop through the esophagus. The crop serves as temporary storage for feedstuff, which is mixed with water. Chickens have a proventriculus (glandular stomach) together with a gizzard (muscular stomach). The proventriculus initiates the digestion of feed by secreting hydrochloric acid and digestive enzymes [16]. Tabata and Yasugi [17] reported that the expression of a spasmolytic polypeptide on the surface cells of the chicken proventriculus plays an important role in the repair of the mucosal epithelium to maintain the status of good gut health. In addition, the proventricular mucus forms a physical barrier to provide protection against pathogens [15]. Beyond this physical barrier, the expression of a moderate level of beta-defensin 6 in the proventricular mucosa indicates a broad-spectrum antimicrobial activity to exclude pathogens [18].

The gizzard, a distinctive feature of the chicken GI tract, is located at the posterior of the proventriculus. Although mainly mechanical digestion occurs in the gizzard, the digestive action of pepsinogen and hydrochloric acid has also been reported in this region, owing to a short retention time of the digesta in the proventriculus [19]. Hydrochloric acid lowers the gizzard pH, which provides a beneficial effect on gut health through disinfection. Gizzard development helps to improve pancreatic enzyme secretion in the small intestine, enhances GI tract motility, and improves nutrient digestibility, thereby enhancing gut heath and function [20].

The small intestine aids in nutrient digestion and absorption with the help of epithelial finger-like projection villi [16]. The breakdown of feed into nutrients takes place in the intestinal lumen with the aid of gastric enzymes secreted by the pancreas and bile secreted by the liver. After digestion, the absorption of nutrients occurs through the wall of the small intestine into the bloodstream. In addition to digestion and absorption, the small intestine also plays a dynamic role in maintaining the gut health of chickens and functions in both innate and humoral immunity in the small intestine [15,21]. Four types of barriers, including microbial, chemical, physical, and immunological, work integrally in the intestine to maintain the intestinal barrier functions to prevent the growth of harmful entities, including pathogens, toxins, and foreign antigens [22,23]. Microbial and chemical barriers are comprised of an outer and an inner mucus (mucopolysaccharide) layer. These mucus layers are secretions from the goblet cells present on the surface of the intestinal villi [24]. The outer mucus layer forms a habitat for commensal bacteria. However, the inner mucus layer, also known as the protected zone, is protected from external microbial exposure of the epithelial cells to remain sterile. An AMP present in this layer limits microbial penetration into the epithelial cells and thereby maintains the gut health of the chicken [25]. Moreover, immunoglobulin A (IgA) and bioactive molecules secreted from the epithelial cell surface into the inner mucus layer reduce the number of pathogens adherent to the epithelia and limit their translocation across the epithelium [22]. The intestinal epithelial cell barrier performs a pivotal role as the first line of defense and as a physical barrier between the host and the luminal environment [23]. It is composed of a single continuous layer of different intestinal epithelial cells bound by tight junctional complexes [22]. These multi-protein tight junction complexes are crucial to maintaining the gut integrity for its proper function. Reduction in the tight junction integrity leads to a leaky gut and allows luminal antigens to enter the body from the mucosa by destroying the gut mucosal homeostasis [26]. Lamina propria, which lies below the epithelial cell layer, is a thin layer of connective tissue consisting of immune cells, such as B cells, T cells, dendritic cells, and macrophages [27]. These immune cells function as an immunological barrier, along with the epithelial cell layer, to maintain the intestinal health of chickens. Detailed immune mechanisms mediated by gut lymphoid tissues are discussed further in the later section on “Intestinal immune system development and its role in gut health” in this review.

Fermentation of the fiber or undigested nutrients in the digesta takes place in the caeca, with the aid of microbes, to produce volatile fatty acids in chickens [28]. Chicken caeca possess both innate and humoral immune functions similar to the small intestinal segment [15]. It also plays a prominent role in gut health by providing a niche for large microflora, including zoonotic bacteria [29]. The proximal part of the caeca contains lymphatic tissues, which form a tonsil [30]. The presence of lymphoid nodules throughout the mucus membrane provides evidence of a greater immune defense mechanism in the chicken caeca to maintain gut immune homeostasis. The large intestine or colon is relatively shorter in chickens in contrast to mammals. Moreover, chicken large intestine is also comparatively shorter than its small intestine. It is still to be determined whether the large intestine in chickens plays a similar role in maintaining gut health as the upper segments of the intestine.

### 3.2. Gut–Brain Axis and Gut Health

**Figure 1:**
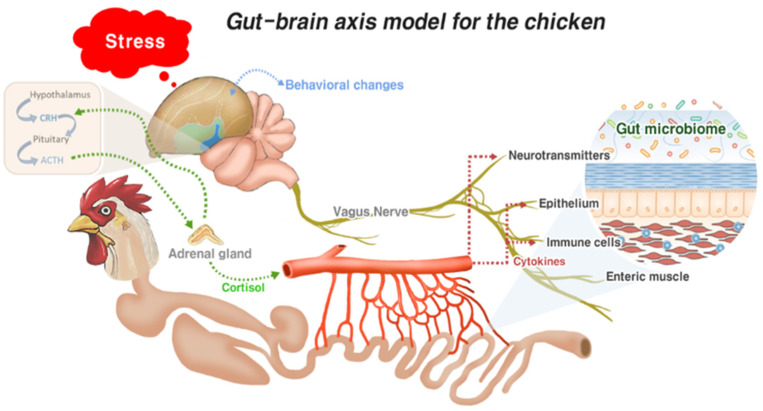
Proposed gut–brain axis model for chickens. The gut–brain axis refers to the bidirectional communication system between the central nervous system and the GI tract. When chickens encounter enteric stress or inflammation, the intestinal epithelium, enteric muscles, and associated immune cells transmit signals to the brain via the central nervous system (vagus nerve). Further, it induces the leucocytes to release cytokines into circulation from the intestine. Cytokines trigger the activation of the central nervous system. Neurotransmitters produced by chicken gut microbes also induce the central nervous system. All these stimuli from the intestine via the vagus nerve activate the hypothalamic–pituitary–adrenal (HPA) axis and increase the serum corticosterone levels. Corticosterone thereby modulates heterophile migration into the inflammation site of the GI tract to attune inflammation. Activation of the HPA axis leads to sickness behavior in chicken, owing to elevated cortisol secretion.

The gut–brain axis refers to the bidirectional communication system between the GI tract and the central nervous system; it plays a responsible role in mediating neural, immunological, and hormonal signaling [31]. This complex system allows the gut to influence the brain through visceral messages. These visceral messages created by the host or gut microbes interact with the enteric nervous system to transduce signals into the brain. In response, signals from the brain influence the gut functions (motor, sensory, and secretory modalities) and immune function. This dual interaction between the gut and the brain not only influences gut physiology but can also affect local pathological processes. The gut–brain axis system is well-documented in mammals [31,32]. However, limited information on the chicken gut–brain axis undermines our understanding of how the brain influences gut health in chickens. The comparison of mammalian and avian neural structures, together with the gut microbiome, described by Villageliũ and Lyte [33], confirms the existence of the chicken gut–brain axis. Based on the literature, we developed a gut–brain axis model for chickens, as shown in Figure 1. When chickens encounter enteric stress or inflammation, the intestinal epithelium, enteric muscles, and associated immune cells transmit signals to the brain via the central nervous system (vagus nerve). Further, it induces leucocytes to release cytokines into circulation from the intestine [34]. Concurrently, cytokines trigger the activation of the central nervous system. Neurotransmitters produced by chicken gut microbes also induce the central nervous system. In the nervous system, the vagus nerve plays an important role in transducing all the enteric signals to the brain. All these stimuli from the intestine activate the hypothalamic–pituitary–adrenal (HPA) axis and increase the serum corticosterone levels [33]. Corticosterone thereby modulates heterophile migration into the inflammation site of the GI to attune inflammation [35]. Moreover, activation of the HPA axis leads to sickness behavior in chickens, owing to elevated cortisol secretion. A combination of decreased feed intake, weight loss, decreased movement, and increased sleepiness is considered as sickness behavior in chickens [36].

Calefi et al. [12] evaluated the interaction of the gut–brain axis during necrotic enteritis (NE) in broiler chickens and showed that NE significantly changed the behavior of chickens in terms of sleeping, walking, feeding, and standing behaviors. Moreover, NE influenced the expression of C-FOS in the hypothalamus, nucleus taenia of the amygdala, medial preoptic area, and globus pallidus of the chickens [12]. A close interrelation between the gut–brain axis with local gut microbiota affecting chicken gut health has also been recently published [33]. According to Villageliũ and Lyte [33], gut microbiota generates neuroactive compounds that may act locally on the enteric nervous system or are secreted in circulation. Thus, signals may transduce to the brain and influence cognition and behavior or cause changes in the enteric system and provide feedback on the microbiota. Calefi et al. [36] analyzed the neurochemical profile in the hypothalamus, midbrain, and brainstem of broiler chickens exposed to *Eimeria* spp., *Clostridium perfringens* (*C. perfringens*), and heat stress. The findings of this study showed that exposure to *C. perfringens* elevates the dopamine levels in the hypothalamus, rostral pallium, and midbrain. In contrast, exposure to *Eimeria* spp. reduces the dopamine levels in the brain. However, a combination of exposure to both *Eimeria* spp. and *C. perfringens* shows elevated dopamine levels in the rostral pallium and the hypothalamus. These results suggest that *C. perfringens* infection is related to dopaminergic pathway activation in chickens. Moreover, increased intestinal tissue damage caused by a combination of *Eimeria* spp. and *C. perfringens* shows activation of the HPA axis, resulting in increased noradrenaline and norepinephrine concentrations in the central nervous system of the broilers [36,37]. In addition, serotonergic system activation of the chicken midbrain, indicating increased levels of 5-hydroxytryptamine and 5-hydroxyindoleacetic acid, was reported for any type of intestinal infections of chickens [36].

## 4. Intestinal Immune System Development and Its Role in Gut Health

### 4.1. Development of the Chicken Gut Immune System

As the gut is the main portal of entry for pathogenic microorganisms into the deeper body tissues, a sophisticated immune system in the gut is indispensable to protect the host from infectious diseases. Enterocytes are part of the important innate immune system of the intestine, and pathogen-induced local inflammation induces enterocyte differentiation and proliferation in the crypt to replace damaged enterocytes in the villus tip. The majority of the developmental changes in the gut occur during the early days after hatching. The basic structure of the gut, including the crypt-villus, is formed as a result of extensive proliferation and maturation of enterocytes during the first five days after hatching, in which differentiation into mucus-producing goblet cells happened before hatching [38,39]. For 3 to 4 weeks after hatching, the chicken gut develops structurally into sophisticated morphologies, including well-defined Peyer’s patch and cecal tonsils [39]. With reference to the development of immune cells, the innate immune system develops prior to the adaptive immune system associated with T and B cells, and most of the changes related to the diversification of immune cells and the cellular composition of the gut immune system occur around the time of hatching. At the time of hatching, a large number of heterophils and monocytes are found in the blood, but only a few lymphoid cells are found in the intestinal tract. A significant increase in the number of lymphoid cells in the intestine mostly occurs during the first two weeks after hatching and continues to increase until 8 weeks [40]. Lymphocytes are detected in the intestine as early as four days after hatching, and the abundance of lymphocytes in the small intestine occurs earlier than that in the large intestine and cecal tonsils. Proportional changes in the lymphocyte subsets in the intestine increase with the age of chickens and continue until 4 weeks.

In the gut, a highly developed lymphoid tissue, known as the gut-associated lymphoid tissue (GALT), responds to antigenic challenges. The GALT is the largest structure of the immune system in the gut and the primary site of immune induction for appropriate immune responses. The GALT in chickens includes organized lymphoid structures, such as the bursa of Fabricius, cecal tonsils, Peyer’s patches, Meckel’s diverticulum, and lymphocyte follicles, scattered along the intra-epithelium and lamina propria of the gut. Chickens lack typical lymph nodes, which are found in mammals, but they do have a number of lymphoid aggregates that represent the majority of secondary lymphoid tissues. Overall, the gut is populated with heterophils, macrophages, dendritic cells, natural killer (NK) cells, and B and T cells, although the proportions of each cell are dependent on many factors. The bursa of Fabricius is one of the primary lymphoid organs uniquely found in avian species, which is responsible for the development of B cells. The cecal tonsils and Peyer’s patches are defined lymphoid aggregates with a germinal center containing immune cells, such as lymphocytes, plasma cells, and M cells. These cells are responsible for sampling the luminal contents to deliver them towards antigen-presenting cells (APCs), such as dendritic cells and macrophages. Meckel’s diverticulum, a unique lymphoid structure in avian species as a remnant of the yolk in the small intestine, contains a germinal center with macrophages and B cells, although the exact function is still unknown [41]. Within the gut, there are two anatomically different lymphocyte compartments, intraepithelial lymphocytes (IEL) and lamina propria lymphocytes (LPL). IEL are distinct gut structures of the epithelial layers of the gut, consisting of a highly specialized group of lymphocytes. The major subsets of IEL are NK and T cells, expressing γδ T-cell receptors (TCR) or αβ TCR; CD8+ T cells are predominant over CD4+ T cells, whereas B cells and heterophils are very few unless they are activated. LPL are located beneath the epithelial layers and are highly populated with a wide range of different immune cells, including granulocytes, macrophages, dendritic cells, and B and T cells; they comprise a major population of lymphocytes, which account for about 90% of immune cells. The T cells in LPL contain a large proportion of the αβ T-cell phenotype, dominated by CD4+ cells, and have a smaller population of the γδ T-cell phenotype [42,43].

Compared with the gut immune system of older chickens, the gut immune system of young chickens is poorly developed, with full development occurring around 4–6 weeks after hatching. Overall, the chicken gut is a dynamic organ with various immune compartments and constantly undergoing substantial changes before and after hatching under the influence of physiology, microbiota, and diet. Thus, the mechanisms involved in evading infection can be very different between young and old chickens.

### 4.2. Development of Avian T and B Cells

The process for the development of T cells in chickens is similar to that of mammals, with some unique features. In early embryonic development, T-cell progenitors are colonized in the thymus, which is a primary lymphoid organ responsible for T-cell development. All lymphocytes developed in the thymus are bloodborne hematopoietic cells that originate from an extrinsic origin [44]. The colonization of T-cell progenitors occurs in three consecutive waves: the first wave originates from the paraaortic hematopoietic foci beginning at embryonic incubation day (EID) 6, the second originates from the bone marrow at EID 12, and the last wave originates from the bone marrow around EID 18 until just after hatching. Each wave of thymic colonization lasts up to 1 or 2 days, followed by refractory periods when the progenitors are completely absent from the blood [44]. After colonization in the thymus, progenitors start differentiating into T cells expressing a certain type of TCR. The increase of γδ T cells occurs earlier than αβ T cells at an interval of approximately 3 days. For example, differentiation into γδ T cells starts at EID 15, and both αβ T cells, αVβ1 and αVβ2 T cells, appear at around EID 18 [45]. The αβ T cells express both molecules CD4 and CD8 on their surfaces, but they are converted to matured T cells expressing a single cell surface marker, either CD4 or CD8, through clonal selection after expansion. On maturation, T cells travel from the thymus to peripheral organs, such as the spleen and intestine. The migration to the periphery organs occurs in the same order as they are developed in the thymus: γδ T cells migrate first, followed by αβ T cells. In the chicken intestine, particularly in IEL, a large number of both αVβ1 and γδ T cells migrates from the thymus and resides; αVβ2 T cells are rarely found in the intestine [46].

As described above, the bursa of Fabricius is unique to avian species and is responsible for the development of B cells, which occurs in the bone marrow of non-avian species, like mammals. Despite the differences, B cell development in avian species is very similar to that in mammals [47]. The bursa provides a unique microenvironment essential for the proliferation and differentiation of B cells. Chicken B-cell development occurs in three stages: the pre-bursal, bursal, and post-bursal stages. The variable genes of the heavy and light chains of immunoglobulin (Ig) are rearranged at EID 5 before entry into the bursa. The invasion of B-cell progenitors into the bursa occurs between EID 8 and 14 and lasts for a few days. At hatching, the bursa includes about 10,000 follicles, which are equivalent to 109 B cells, with more than 90% of the bursal cells being mature B cells [48,49]. The three major classes of antibodies in avian species are IgM, IgA, and IgY, which are equivalent to IgG in mammals. The lifetime B-cell repertoire is generated in the bursa and is influenced a lot by diet and gut microbiota. The IgM+ B-cell precursors are found as early as EID 12, and IgY+ B cells appear around the time of hatching. The post-bursal stage begins with B-cell migration and lasts until just before hatching. The B cells migrate to the outside of the bursa after development in the order of their maturity, and IgM+ B cells are detected earlier than IgY+ B cells in the periphery. After hatching, the expansion of B cells in the secondary lymphoid organ begins on the 3rd day for IgM+ B cells and the 8th day for IgY+ B cells [50]. Besides the bursa, avian species have a unique organ, the Haderian gland, around the orbit of the eye; it contains a large number of Ig-secreting plasma cells [51]. In contrast to mammals, where the transfer of maternal antibodies takes place via the transplacental and colostral routes, the transfer of maternal antibodies in avian species occurs via egg yolk transfer [52]. Maternal antibodies are secreted into the albumin to be taken up by the developing embryo, and IgY is the major class of antibodies transferred via egg yolk [53]. A high IgY titer is crucial for protection against various pathogens, including coccidiosis, Salmonellosis, and Campylobacteriosis, even though they do not circulate in young chickens for a long time [54,55]. The level of maternally derived antibodies wanes after three weeks of age as the B cells of young chickens start producing their own antibodies.

### 4.3. Inflammation in the Gut

Since the gut is constantly challenged by the antigenic substances from the diet and the dynamic ecosystem of gut microbiota populations, inflammation, which is an important part of innate and adaptive immune responses to protect the host against antigenic challenges, plays a critical role in the healing process and dictates the status of the gut health. Although inflammation is a vital response to infections for the survival of the host, the balance of factors that promote and regulate inflammatory responses needs to be better understood. In livestock, inflammation that is initiated by diet or external stressors often causes dysbiosis, with unwanted consequences on growth performance and gut health [56]. Thus, studies on effective methods for chicken production are directed towards balancing pro- and anti-inflammatory responses to prevent overzealous chronic inflammatory responses that can be detrimental to gut health. The main limitation of an anti-inflammatory approach in chickens is their vulnerability to infections, particularly in young chickens, since their immune systems are not fully developed. Gut inflammation is initiated by gut resident immune cells, mostly innate immune cells, including APCs and heterophils, that express pattern recognition receptors (PRRs). In the chicken gut, various toll-like receptors (TLRs), including TLR1, 2, 3, 4, 5, 7, and 15, are expressed [57], and the activation of TLRs induces downstream signaling pathways, such as mitogen-activated protein kinase (MAPK) and nuclear factor kappa beta (NF-κB). This results in a protective immune response supported by the recruitment of immune cells and the expression of associated proinflammatory mediators and AMPs. The process of inflammation consumes nutrients, which are routed from productive purposes, thus reducing the appetite in poultry. Therefore, it is critical to find a point of balance between inflammation and anti-inflammation for a healthy gut in poultry.

### 4.4. Mechanism of Immune Responses in the Gut

GALT plays a major role in the gut immune response, inducing a full spectrum of innate and adaptive immune responses that vary depending on the nature of the stressors, including pathogens. The common features of the host immune response aim to limit the infection. The mucus layers, with varying thicknesses and compositions, trap invasive bacteria via the luminal flow, as well as providing lubrication, and colonization sites, nutrients for commensal bacteria, and a transport system between the gut contents and epithelial linings [58]. The optimum mucus layer thickness is represented as the number of goblet cells secreting mucins. Mucins can be either neutral or acidic, and the latter protects against bacterial translocation [59]. Abnormal mucus layer thickness is associated with enteric infection and poor performance [60]. Besides the mucus layer, AMPs and secretory IgA (sIgA) are integral components of the gut immune system. AMPs are expressed by a variety of cells, including Paneth cells. Defensins are the most studied AMPs in chickens; the chicken genome encodes for 14 β-defensin genes, also known as gallinacins, but no α-defensins [61]. NK-lysin, a homolog of human granulysin, has also been reported to play a role in gut infection by protozoan parasites such as *Eimeria* spp. [62,63,64]. They possess broad-spectrum antimicrobial activities against Gram-negative and Gram-positive bacteria, fungi, viruses, and protozoa [65]. They are also involved in the modulation of immune responses and determination of the microbiota composition [61]. IgA is secreted from the plasma cells present in the lamina propria; they can neutralize pathogens and facilitate their removal from the GI tract and play a role in intestinal homeostasis, including establishment, maintenance, and the control of commensal microbes [66]. Since lamina propria IgA-positive cells and intestinal IgA levels are both significantly reduced in germ-free animals, they could play a potential role in the gut microbiota [67]. Several studies have been conducted on the relationship between gut microbiota and the host immune response. The mucus secreted from goblet cells can act as a source of nutrients for the resident microbiota, which can be used to inhibit the expansion of other bacteria [68]. Controlled inflammation requires gut microbiota to regulate the gut immune response either towards inflammation or tolerance [69].

## 5. Microbiota and Its Role in Chicken Gut Health

The chicken gut is home to more than 100 trillion microorganisms collectively called the gut microbiota, whose membership includes bacteria, fungi, protozoa, and viruses [70]. Among these microbes, bacteria play a dominant role in intestinal functions. They are colonized in different segments with vast diversities; they help in digestion, developing the local immunity, and intestinal health.

The crop and gizzard are usually dominated by *Lactobacilli* and *Streptococci*, accounting for 10^3^–10^4^ colony-forming units/g [71]. Compared to the rest of the small intestine, the ileum contains the richest bacterial community, whereas the duodenum has the lowest bacterial density [72]. The majority of the microbiota community in the duodenum are *Lactobacilli* and *Bifidobacteria* [73]. *Lactobacilli, Enterococci,* and *Clostridiaceae* are commonly detected in the jejunum [74], while *Streptococci, Bacteroides, Clostridia, Escherichia coli, Ruminococci, Enterococci,* and *Lactobacilli* are dominated in the ileum [75]. In comparison to the ileum, the ceca harbor a more diverse, abundant, and stable microbial community that is composed of the genera *Bacillus, Clostridium, Enterococcus,* and *Ruminococcus* [76,77]. Similar to the caeca, the cloaca also shows a more diverse and abundant bacterial population, consisting of *Clostridia, Streptococci, Enterococci,* and *E. coli* [75].

### The Role of Gut Microbiota in Chicken Gut Health

One of the key functions of the gut microbiota is extracting essential nutrients from the diet and making them available to the chicken [78]. This involves the extraction, absorption, and synthesis of a wide variety of biochemicals with growth-promoting effects, including water- or lipid-soluble vitamins (e.g., vitamin B and vitamin K), organic acid (lactic acid), complimentary enzymes (e.g., non-starch polysaccharides (NSPs)), and antimicrobial compounds [79]. Soluble dietary fibers and resistant starch are actively fermented by cecal microbiota to produce metabolites such as short-chain fatty acids (acetate, butyrate, and propionate), which are beneficial to the physiological functions of chickens [80]. The intestinal microbiota significantly influences the drug metabolism [81] and nitrogen metabolism [82]. Thus, an imbalance in the microbial community affects gut microbial fermentation, host metabolism, and gut health.

As mentioned earlier, the gut microbiota produces metabolites and influences intestinal homeostasis [83]. Maintaining a balanced homeostasis supports the tolerance for infections and nonpathogenic stressors [84]. Metabolites, mediated by the microbiota, are regarded as major modulators of host-microbiota communication and closely interact with the host immune system, which, along with diverse types of immune cells, mediates various immune signaling pathways [85]. The concentration, composition, and the type of metabolites connected with the host sensor molecules lead to the immune function regardless of the steady state or disease duration [86]. Therefore, well-balanced metabolites influence the immune homeostasis and influence the gut health and growth of chickens. Park et al. [87,88] reported that chickens fed a diet supplemented with growth-promoting probiotics or phytochemicals showed the significant alteration of many gut metabolites in the ileum, whose functions are associated with host immunity, gut integrity, and muscle growth.

The gut microbiota is also involved in protecting gut barrier functions to improve gut health. The gut microbiota protects the intestinal barrier by attaching to the epithelial walls of the enterocyte and reducing the colonization of pathogenic bacteria [79]. Simultaneously, the beneficial bacteria, including *Lactobacillus plantarum, L. reuteri,* and *L. rhamnosus*, help the development of the mucosal epithelia, especially immune cells whose primary function is to protect the gut barrier against pathogens [74]. The inner layer of the mucus functions as a highly efficient first line of defense against pathogenic microbiota, such as *Clostridium jejuni* and *Helicobacter pylori* [89]. Sommer et al. [90] reported that the intestinal microbiota has a significant impact on the mucus layer based on the observation of fewer goblet cells in germ-free mice. Moreover, the commensal microbial population works against the pathogen invasion through competitive exclusion, thus maintaining gut homeostasis [72].

## 6. Intestinal Infections and Their Impact on Gut Health

**Figure 2:**
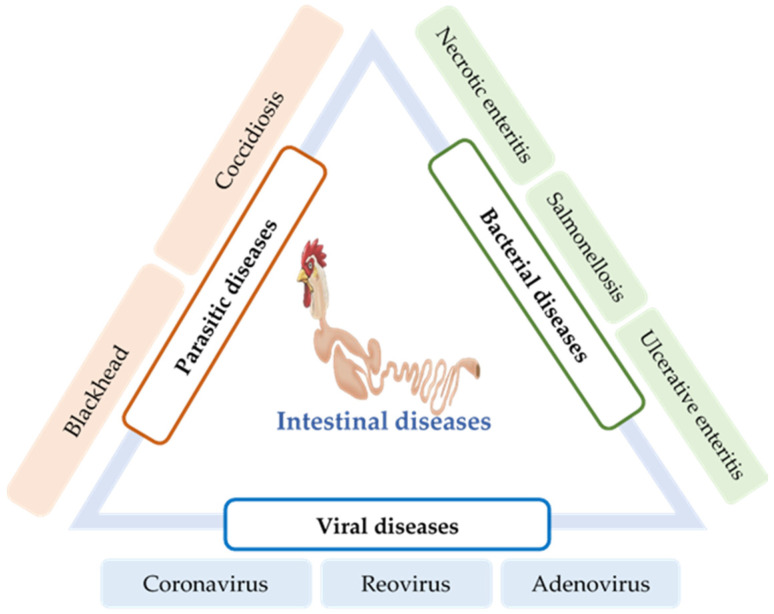
Major intestinal infectious diseases in poultry. Based on the nature of the causative agent, intestinal infections can be divided into three categories: parasitic, bacterial, and viral diseases.

Intestinal infection caused by parasites, viruses, and bacteria is a major factor that causes dysbiosis and disturbs the intestinal immune homeostasis. Some of these diseases are the results of secondary infections and may involve respiratory tracts and other organs. Based on the nature of the causative agent, intestinal infections can be divided into three categories: parasitic, bacterial, and viral diseases (Figure 2).

### 6.1. Parasitic Diseases

#### 6.1.1. Coccidiosis

Coccidiosis is a common intestinal disease caused by several species of the protozoan parasite of the genus *Eimeria*, which invades the intestinal lining of chickens. It is one of the major intestinal infectious diseases of poultry that causes severe economic losses worldwide [91,92]. At least seven species of *Eimeria* (*E. acervurina, E. maxima, E. tenella, E. mitis, E. necatrix, E. brunetti, and E. praecox*) have been recognized based on molecular characteristics [93]. Among these, *E. maxima* and *E. tenella* are the most prevalent and pathogenic species that cause the highest economic impacts on the chicken industry [94].

Coccidiosis is initiated when chickens ingest the environmentally resistant *Eimeria* oocysts. These *Eimeria* spp. characteristically infect a specific site of the intestinal tract, resulting in severe inflammation and necrosis in the submucosa, although the mechanism of site-specificity of *Eimeria* is not well-known [43,95]. For every oocyst ingested, innumerable parasites are reproduced in the intestine and transmitted to the entire poultry house via contaminated feces. The invasion of these *Eimeria* sporozoites into the intestinal tract and their subsequent intracellular development damages the intestinal epithelium, obstructing nutrient absorption and immune suppression of the host chicken for their survival. Consequentially, poor growth performance and higher mortality have been reported in chickens [96].

#### 6.1.2. Blackhead

Blackhead (Histomoniasis) is a chicken gut disease caused by the single-celled protozoa *Histomonas meleagridis*. Although it is relatively common in captive-raised game birds and turkeys [97], it is also found in several other avian species, including grouse, quail, and pheasants [98]. The pathogenesis of *H. meleagridis* begins with the colonization of the caecum of the birds, thereafter leading to severe intestinal inflammation and necrosis [99]. *Histomonas* parasites penetrate the blood vessels and reach the liver via the portal veins [97]. Moreover, unusual lesions have also been reported in other visceral organs of turkey, such as the kidneys, lungs, and bursa of Fabricius [100]. In chickens, lesions are mostly found in the caecum, with less or no necrosis in other organs [101].

In turkeys, clinical signs are observed 1–3 weeks following the ingestion of *H. meleagridis*; the symptoms include ruffled feathers, drooping wings, apathy, and sulfur-colored diarrhea [102].

### 6.2. Bacterial Diseases

#### 6.2.1. Necrotic Enteritis

NE is an enteric disease of poultry caused by *C. perfringens*, and coccidiosis is its primary risk factor. It is the major cause of economic losses in the poultry industry, with an estimated annual loss up to US $6 million [103]. *C. perfringens* is a spore-forming, anaerobic Gram-positive bacilli that is widely distributed in freshwater or soil. It is also found as a member of the normal intestinal flora of birds [104]. *C. perfringens* strains are classified into five toxin types (A–E), according to the toxins they produce [105]. B-like toxin (NetB) produced by *C. perfringens* is identified as the causative agent of NE. However, a simple infection is not sufficient to provoke the disease. Increased consumption of barley, wheat, or other poorly digestible proteins and coinfection by *Eimeria* spp. are various predisposing factors that trigger the disease [106,107]. Physiological stress has also been reported as a factor triggering subclinical NE [108]. Broiler chickens are more susceptible to NE; nevertheless, the disease has also been found in commercial, backyard or free-range turkeys, and layers [109,110]. Birds exposed to *C. perfringens* showed impaired weight gain and an increased density of *C. perfringens* in the small intestine [108]. NE in chickens has caused economic losses due to higher mortalities, a poor growth rate, and a lower feed conversion ratio [111].

In several studies, *C. perfringens* exposure has been shown to decrease the population of *Lactobacillus* in the ileum [112,113]. *Lactobacilli* are bacteria producing lactic acid that protect the intestinal epithelium and immune cells from intestinal pathogen invasion and prevent gastric mucosal lesion development. In a recently published study [114], coinfection with *C. perfringens* and *Eimeria* spp. showed a reduced population of *Lactobacillus* and severe NE lesions in the jejunum in chickens.

#### 6.2.2. Ulcerative Enteritis

Ulcerative enteritis (UE) is one of the most common acute bacterial diseases in quail caused by the bacterium *Clostridium colinum*. This disease has also been reported in chickens, turkeys, and pheasants [115,116]. Compared with older birds, young birds, in the age group of 4–12 weeks, are most susceptible to this disease. In young quails, their mortality may be as high as 100% within a few days [117]. Several predisposing factors, such as coccidiosis, chicken infectious anemia virus (*circovirus*), Gumboro disease, and stress conditions, may increase the incidence of UE and subsequent mortality [118]. Intestinal lesions are characterized by multiple ulcers throughout the tract, including the duodenum, jejunum, ileum, and cecum; peritonitis and multifocal necrotizing hepatitis are also observed in many cases [119]. However, limited studies with UE have been reported, since few avian species and other commercial chickens have been affected by UE.

#### 6.2.3. Salmonellosis

Salmonellosis is a collection of infectious diseases caused by the species belonging to the genus *Salmonella*. Imbalance in the normal intestinal microflora due to antibiotic abuse, deficiency of nutrients, and *Eimeria* infections has been associated with *Salmonella* proliferation and colonization in the GI tract [120].

In chickens, enteric lesions induced by *Salmonella* infections are often associated with secondary infections with fowl typhoid (FT) and Pullorum disease (PD). FT and PD are the most significant bacterial diseases in chickens [121]. FT is an acute or chronic septicemic disease of mature chickens caused by *Salmonella Gallinarum*, whereas PD, caused by *Salmonella Pullorum*, is an acute systemic infection occurring more in young broiler chickens. In the early 1900s, both of these infections recorded high mortality (up to 100%) that led to serious economic losses of the poultry industry [122]. PD and FT follow various routes of transmission, but mostly, they are transmitted orally via diet, water, cannibalism, and, often, by the respiratory tract; the causative agents may also enter the body through wounds. Transovarian infection, resulting in infection of the egg, has also been reported [123]. *Salmonella* asymptomatically infects the ovaries of hens, contaminating the eggs inside the chicken even before shell formation. Although, initially, only a small number of eggs may be infected, horizontal transmission can spread the occurrence after the chick’s hatch.

### 6.3. Viral Diseases

#### 6.3.1. Coronavirus

The poultry coronavirus, also known as the infectious bronchitis virus (IBV), is a highly contagious, acute disease-causing agent of poultry. High mortality is the major concern of coronavirus infection. However, poor growth performance, impaired feed efficiency, renal disease, insufficient egg quality, and decreased egg production are other issues associated with IBV infection [124]. Although IBV mainly replicates in the epithelial surface of chicken respiratory tracts and causes respiratory illness, it can also replicate in the enteric tract, oviducts, and the kidneys [125,126]. However, the replication of IBV in the chicken intestine does not cause any pathological changes [127]. The susceptibility of birds to the IBV strains is triggered by various factors, including environmental stress, age, and genetics [128]. The domestic chickens and pheasants (*Phasianus* spp.) are primarily the susceptible hosts for IBV [129].

#### 6.3.2. Reovirus

Avian reovirus (ARV) is a nonenveloped, double-stranded RNA virus belonging to the genus *Orthoreovirus* in the *Reoviridae* family [130]. ARV is ubiquitous among commercial poultry and has been reported to be responsible for a variety of disease conditions in poultry, including malabsorption syndrome (MAS), runting-stunting syndrome (RSS), tenosynovitis, gastroenteritis, and immune suppression [131,132,133]. The most common mode of transmission of ARV is the fecal–oral route; following which, the initial replication occurs in the mucosa of the intestinal and respiratory tracts. However, infection via egg transmission has also been reported [134,135]. Most of the studies establishing ARV pathogenesis have implicated that the intestinal tract is a significant region of ARV infection, regardless of the route of inoculation [136]. Generally, ARV initially replicates in the villus epithelium of the small intestine and the bursa of Fabricius and subsequently disseminates to other organs [137]. ARV infection makes birds vulnerable to infection from other pathogenic agents by inducing immune suppression.

#### 6.3.3. Adenovirus/Hemorrhagic Enteritis (HE)

Adenoviruses are nonenveloped, icosahedral viruses that belong to the family *Adenoviridae*. Adenoviruses can be subdivided into a mammalian adenovirus (mastadenovirus) and avian adenovirus (aviadenoviruses) [138]. Further, avian adenoviruses can be subdivided into groups I, II, and III. Group I avian adenoviruses are usually found in excreta or in the tissues of birds with GI diseases. Various diseases, including runting/MAS [139], proventriculitis [139], ventriculitis [140], enteritis [141], and mortality syndrome [142], are associated with group I.

HE infection induces an immunosuppressive response; hence, it can increase the risk of secondary infections by bacterial diseases. Turkey is the most susceptible to HE infection, although serological evidence suggests that commercial chickens can also be prone to HE infection [143]. The transmission of a HE-causing virus primarily occurs by the fecal–oral route. The virus can survive for several weeks in contaminated litter, in carcasses protected from drying, or in wet fecal material [132]. During the infection, the proximal small intestine (duodenal loop) is typically distended, grossly discolored, and filled with blood-mixed exudate. In severe cases, expanded lesions are also found in the caecum [144].

## 7. Factors Affecting Intestinal Health

**Figure 3:**
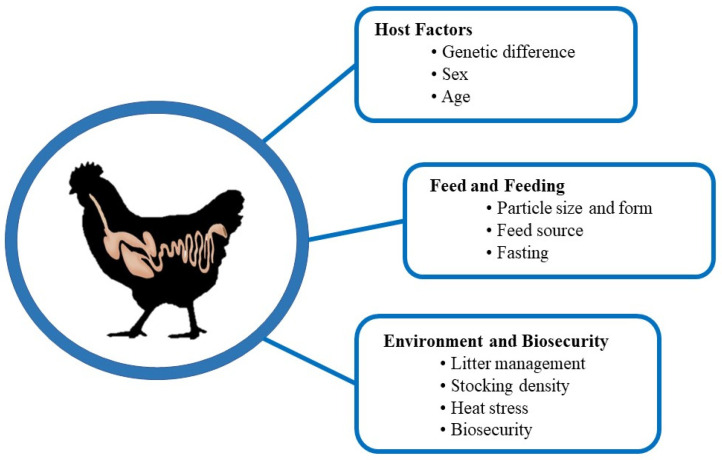
Factors affecting chicken gut health. A wide range of factors affecting chicken gut health can be categorized into three categories: host factors, feed and feeding factors, and environment and biosecurity factors. A single factor or many factor combinations can significantly affect the structure and physiological function of the chicken gut, altering the gut health and performance.

A wide range of factors affecting chicken gut health has been identified. Understanding their mechanisms is critical to maintaining a healthy chicken flock in a profitable way. Different researchers have categorized these factors in various ways [79,145]. Here, we have categorized the factors affecting chicken gut health into the following three categories: host factors, feed and feeding factors, and environment and biosecurity factors (Figure 3).

### 7.1. Host Factors

#### 7.1.1. Genetic Difference

The genetic makeup of chickens influences many aspects of the gut health, including the host immune response and disease susceptibility [25,107]. Indeed, altered gut health, with respect to the gut microbiome and local immunity, and gut morphology among genetically different chicken breeds has been demonstrated [145,146]. Chicken breeds with different genetic backgrounds have been known to be one of the key factors affecting host immune responses to pathogen exposure in chickens [42,147,148]. Han et al. [149] reported that, compared to broiler chickens (Ross 308), layer-type chickens (Lohmann leghorn) possess a higher percentage of CD4+ and CD8+ in lamina propria lymphocytes in the cecum. In addition, the higher gene expression of proinflammatory cytokines (i.e., interleukin IL-6 and IL-8) in the ceca was shown in layer chickens compared to broiler chickens. This may explain different gut health status between broiler and layer chickens that have undergone genetic selection based on growth performance (for broilers) and reproductive performance (for layers). It should be noted that commercial broiler lines (e.g., Ross, Cobb, and Hubbard), which were selected by growth performance criteria, exhibit altered gut physiology and the gut microbiome. Jang et al. [150] and Kim et al. [146] reported that commercial broiler chicken lines (Ross, Cobb, and Hubbard) show different disease susceptibility patterns upon exposure to gut-specific pathogens. For example, compared to Ross and Hubbard, Cobb exhibits greater disease susceptibility with regard to gut lesions and body weight loss over the course of pathogen exposure. Kim et al. [146] further demonstrated a role of ileal gut microbiota associated with different disease susceptibility of these three commercial broiler chicken lines before and after pathogen exposure. Dietary phytochemicals affect the genetically determined ileal microbial profiles, indicating that broilers with different genetic backgrounds exhibit different biological responses to nutritional intervention [25,151]. Furthermore, Ross broiler chickens, compared to Cobb, exhibit higher expression patterns of genes encoding for host beta-defensins. Hong et al. [25] postulated that differences in gene expression levels, including that of beta-defensins, might explain the disparate disease resistances or susceptibilities among broiler chicken lines. In many broiler breeds that have been selected based on performance criteria, it is evident that host genetic factors play a critical role in determining their disease susceptibility to enteric diseases, including NE [148].

Adeleye et al. [152] reported that the villus height and crypt depth of the small intestine varied between two strains of broiler chickens (Arbor acre and Marshal), although they were both raised under similar environment and management conditions, including diet and floor bedding. However, no consistent trends were noted with the strain-specific development of morphological characteristics in the duodenum, jejunum, and ileum. In general, genetically selected broiler lines (i.e., a commercial meat-type chicken broiler line) tend to have bigger crypt sizes and greater enterocyte migration rates within the villus than do less- and non-selected chicken lines [153]. However, genetic selection does not affect the density, dimensions, or pattern of enterocyte microvilli. Similarly, during incubation and 7 days post-hatch, fast-growing broilers have a greater villus surface area and an elevated number of enterocytes per villus than that observed in slow-growing layer chickens [154,155]. Zulkifli et al. [156] also noted that, compared to red jungle fowl, broiler chickens have a greater villus height and width of the duodenum, ileum, and ceca. They also reported that, compared to red jungle fowl, broiler chickens have more *Lactobacillus* spp. in the ileum and *Streptococcus* spp. in the ileum and cecum. Thus, genetic selection for growth tends to equip the greater absorptive capacity of the small intestine, facilitating the quantitative and qualitative absorption of nutrients.

Sun et al. [157] analyzed the gut microbial composition of two native chicken breeds, Caoke chickens and Partridge Shank chickens; Caoke chickens are more resistant to disease than Partridge Shank chickens. However, no specific pathogen was described. It was observed that, compared to Partridge Shank chickens, Caoke chickens showed a higher percentage of the phyla *Firmicutes* but a lower percentage of the phyla *Bacteroidetes* in the cecal contents. However, Sun et al. [157] specified that microbial colonization is more affected by the environment that the chickens were raised in, such as the housing system, than the breed. Recently, clear differences in the intestinal morphology and tight junction proteins between commercial and native chickens were addressed [158]. It was noted that, compared to native Thai chickens, broiler chickens (i.e., Arbor Acres) have increased villus length: crypt depth ratio, number of goblet cells, and β-defensin-positive Paneth cells in all the segments of the small intestine. In addition, an immune-histochemistry study revealed that tight junction-related proteins (i.e., claudin-1 and occludin) are detected frequently in all the segments of the small intestine of broiler chickens than in Thai native chickens. Collectively, these variations by genetic background indicate that the continuous selection for growth and/or body weight has led to changes in microbial ecology and the function of the GI tract in chickens.

#### 7.1.2. Sex

Due to the typical production system for the broiler and egg industries, sex as a confounding factor in gut health for broilers and laying hens is often neglected. However, Kers et al. [145] emphasized that sex is one of the important factors affecting the development of gut microbiota in chickens, since less than 30% similarity in the microbial community has been observed between sexes, based on a denaturing gradient gel electrophoresis analysis [159]; the clear difference in the abundance of *L. salivarius, L. crispatus, L. aviaries,* and *E. coli* in the cecal contents has been observed between sexes [160]. In addition to the disparity in the gut microbiota between male and female chickens, it was postulated that differences in the growth and feed conversion ratio could have a distinct impact on gut health. In addition, at 35 days, a difference in the cecal microbiome between male and female chicks of the same broiler line was reported [161]. Lee et al. [161] found that male chickens enriched more frequently with *Bacteroides*, while female chickens showed enrichment of *Clostridium and Shigella.* Regarding gut morphology, it has been reported that, compared to female chickens, male chickens have lighter and shorter small intestine per 100 g body weight [162,163]. In contrast, compared to female chickens, male chickens have longer villus height in the duodenum, thus increasing the surface area for nutrient absorption, but this was not detected in the ileum [162]. Collectively, these findings may suggest gender-associated differences in the gut microbiota and gut structure. This suggestion implies the possibility of the susceptibility of gender to specific enteric diseases, including avian coccidiosis. According to the study by Lu et al. [164], the *Eimeria* challenge increases the concentration of sIgA in the jejunal mucosa, independent of the sex. However, the jejunal villus height and villus height and crypt depth ratio, but not crypt depth, tend to be higher in male broilers than that in female broilers challenged with *Eimeria*. In addition, Lu et al. [164] also found that male broilers tend to have less cecal gut damage upon exposure to *Eimeria tenella* than female broilers, indicating a resistance to innate coccidiosis [165].

#### 7.1.3. Age

It is now well-established that gut microbiota induces host immune maturation [166]. Using germ-free chicks, it was found that the absence of gut microbiota or heat-killed microbiota failed to induce the expression of immunoglobulins in the cecum of 56-day-old germ-free chickens, indicating the role of viable gut colonization on immune development even at a later stage [167]. Thus, it is expected that the early establishment of gut microbiota in chickens would help the chickens to develop immune maturation. Ding et al. [168] found that 65 genera can be considered as core microbes that exist in the embryos, chicks, and maternal hens, such as *Halomonas, Lactobacillus, Bacteroides,* and *Enterococcus.* The study by Roto et al. [169] implied that the microbiome present in the oviduct of breeder hens would affect the establishment of the gut microbiota in the progeny chicks, which rendered the scientific basis of the in ovo technique of single or mixed bacteria for the development of optimal gut health. However, it is to be noted that there is age-specific development of the gut microbiota, which is not entirely attributable to environment modification. For example, Donaldson et al. [170] found that the administration of cecal contents, obtained from adult healthy broiler chickens to incubating eggs, did not affect the gut microbiota of hatched broiler chickens. In contrast, all chickens exhibited the age-dependent development of gut microbiota: *Faecalibacterium* increased in abundance slowly and steadily with age, the abundance of *Lactobacillus* did not change over the course of the study, and *Enterobacter* was only abundant in the early days of life after hatching. In a study by Jurburg et al. [171], three successive stages in the fecal microbiome of male broiler chickens were detected. They monitored the fecal microbiome of male broilers from days 1–7 and days 10, 14, 21, 28, and 35; they found the age-dependent microbiota patterns as vertically transmitted taxa, including *Streptococcus* and *Escherichia/Shigella*, on day 1, followed by rapid-growing taxa, including *Lachnospiraceae* and *Ruminococcus*-like species on day 4, and, finally, slow-growing taxa, including *Candidatus, Arthrobacter,* and *Romboutsia*, on day 10. The optimal times for microbiome interventions between successional stages were found to be 3 to 4 and 14–21 days after hatching.

### 7.2. Feed and Nutrition

In modern poultry production, commercial broilers and laying hens, upon hatching, are provided with an ad libitum diet. Thus, diet is considered one of the most important factors directly or indirectly affecting the balance between microbiota and the gut health. In the chicken industry, it is common to use a less expensive feed formulation to produce more meat and eggs at the least possible feed cost, targeting the production of low-cost, high-quality finished products. Due to the importance of gut health, the effect of feedstuffs or feed forms on the microbiota and health of the gut has gained prominence.

#### 7.2.1. Particle Size and Form of Feed

It is known that both feed grinding (finely or coarsely ground feed) and feed processing (mash or pellets) improve the quality and safety of feed and are known to affect the feed efficiency upon ingestion [172,173]. Consequently, the grinding and pelleting of feed influence the gut microbiota and intestinal contents of volatile fatty acids in broilers [174]. The fine grinding of feed increases its surface area but is known to exhibit a shorter digesta transit time than that of coarsely ground feed and also delays gizzard development [175]. Thus, it has been accepted that coarsely ground feed in the diet of chickens facilitates the development and functionality of gizzards, which also influences the intestinal morphology and functionality [172]. Similarly, Kheravii et al. [176] observed that, compared to finely ground corn, coarsely ground corn increases the relative gizzard weight and upregulates genes-expressing digestive enzymes (e.g., aminopeptidase in the duodenum) and nutrient transporters (alanine, serine, cysteine, and threonine transporter-1 in the jejunum) in broilers. Researchers speculate that enhanced gizzard function due to feeding them coarsely ground corn could stimulate gut motility, including active reverse peristalsis and a longer digesta retention time, leading to the enhanced production of digestive enzymes and nutrient transporters.

Compared to mash, the pellet is known to increase the growth of chickens via increased feed intake but not the lower relative weight of the gizzard [174]. Similarly, the relative empty weight of the gizzard was greater in chickens fed a mash diet than those fed a pellet diet [177]. Amerah et al. [177] concluded that, compared to the particle size (fine or coarse), the feed form (mash or pellet) is a more significant decisive factor influencing the development of the digestive tract in chickens. This indicates that pelleting itself equalizes the effect of the feed particle size, as it has softening (via steaming) and grinding (via pellet press) effects during the pellet manufacturing process. It is also expected that the feed form could affect enteric pathogens or gut microbiome in chickens, as it affects the dynamics of gut digesta and development. Compared with mash-fed chickens, broiler chickens fed a pellet diet had a greater abundance of *coliform* bacteria and *enterococci* in the ileum and a reduced number of *C. perfringens* and *Lactobacilli* in the ceca and rectum. In addition, the volatile fatty acid content in the ceca of pellet-fed birds was significantly higher than that in mash-fed birds [174]. Although the pellet diet increases the beneficial bacteria and volatile fatty acids in the GI tract, ironically, pellet-fed broilers are more susceptible to *Salmonella typhimurium* infection than the mash-fed counterparts [178]. This study emphasizes that the fully developed gizzard, which can be accelerated using a feed size or processing method, might play an important role in controlling avian pathogens in chicken digesta.

Regarding the gut morphology, chickens fed the pellet diet showed higher villus heights and villus height-to-crypt depth ratios than those fed the mash diet. The extended villi in villus morphology could possibly be the physical response to increasing the absorptive surface to maximize the digestion and absorption of the nutrient overload by an increased feed intake [172]. Similarly, Abadi et al. [179] found that pellet-fed broilers have longer duodenal villus heights but a lesser number of goblet cells in the duodenum and lesser ileal crypt depths than those in mash-fed broilers. It was speculated that pellet-fed chickens might have a lower demand for mucin production due to the reduction of harmful bacteria in the feed during the pelleting process. Both the feed form and feed particle size did not affect *Coliform* spp. and *Clostridium* spp. in the cecal digesta. However, compared with finely ground feed, coarsely ground feed increases the abundance of lactic acid bacteria spp. in the cecal digesta. In summary, in addition to the significant impact on growth performance, it is likely that both feed processing and feed grinding influence the intestinal morphology and microbiota of chickens via improved gizzard function. It has been proposed that relative gizzard weights are an important indicator of gut integrity [180].

#### 7.2.2. Feeding Stuff

The presence of antinutrients and varying nutrient compositions in feedstuffs and the undigested feed origin nutrients available for distal fermentation can affect the gut microbiota, which further influences the development of the intestinal microstructure and microbial ecosystem. Lourenco et al. [181] reported that chickens fed a soybean-based diet have a higher abundance of *Campylobacter* than those fed a soy-free diet. Researchers believe that the presence of NSPs in soybeans and their effect on mucin production might play a role in altering the levels of mucin and short-chain fatty acids, which increases the birds’ susceptibility to *Campylobacter*. Peas are a traditionally grown protein source rich in essential amino acids and starch; their use for poultry feeds is limited due to the presence of various antinutrients, including antigenic proteins, lectins, and NSPs. Röhe et al. [182] found that feeding raw peas lowers the villus surface area of the jejunum than feeding them with a soybean meal diet. In addition, compared with the soybean meal, the dietary pea meal downregulates the relative mRNA expression of the genes related to glucose transport in the jejunum (e.g., sodium-dependent glucose co-transporter 1 and glucose transporter 2) but does not affect the intestinal permeability (i.e., zona occludens (ZO)-1 and claudin 5) and cell maturation (alkaline phosphatase and caspase 3) in the jejunal tissues. Röhe et al. [183] found that broilers fed a diet containing peas have immunohistochemically higher numbers of intraepithelial CD3+ and CD45+ lymphocytes in the tip and mid-region of the jejunal villi than those fed a soybean meal-based diet. However, Röhe et al. [183] failed to observe the differences in the mRNA expression patterns of the proinflammatory and anti-inflammatory cytokines in jejunal tissues of broilers fed with pea and soybean meal-based diets. Thus, two studies [172,183] provided some supporting evidence that the antinutrients present in feedstuffs might inhibit the growth of chickens, but they could augment local immune reactions without affecting the gut integrity.

It is known that fatty acids, especially omega-6 and -3 fatty acids, are highly associated with mucosal immune responses, epithelial barrier functions, oxidative stress, and inflammatory reactions, since they can be incorporated into enteric tissues and cell membranes [184]. In addition, coconut oil, which is a rich source of medium-chain fatty acids, is also known to affect the gut health, owing to its antimicrobial activity [184,185]. Knarreborg et al. [186] reported that the number of *C. perfringens* in the ileal digesta is lower in broilers fed soybean oil than those fed animal fats. It was reported [187] that dietary lipids (lard, linseed oil, and sunflower oil) alter the fatty acid composition and nutrient transporters of the brush border membrane of the jejunum but do not affect the relative weight and villus surface area of the small intestine. Similarly, dietary fats (e.g., sunflower oil) rich in unsaturated fatty acids increase the villus height of each segment of the small intestine more than saturated fatty acids (e.g., palm oil) [188]. Dietary fats, which differ in the ratios of omega-3 and omega-6 fatty acids, also affect the gut health of chickens [189]. Based on an alkaline comet assay analysis, diets with high omega-3 fatty acids or a low ratio of omega-6:omega-3 fatty acids increase the DNA damage of the jejunal tissues of chickens compared to those with high omega-6 fatty acids or a high ratio of omega-6:omega-3 fatty acids. In contrast, feeding oxidized fats has been shown to elevate the intestinal epithelial turnover rates, cause apoptosis at villus tips, and lower the concentration of secretory IgA in the intestinal tissues, thus compromising the gut health [190]. It was also reported [191] that increasing the levels of oxidized fat decreases the mRNA expression levels of claudin-1 and occludin in the ileum of chickens. In contrast, increasing the oxidized fat content in the diet of broilers increases the mRNA expression levels of IL-22 in the ileum [191]. Based on these findings, it is postulated that oxidized fats increase the intestinal permeability, leading to intestinal barrier dysfunction and inducing an inflammatory response (e.g., IL-22 is a proinflammatory cytokine that is predominantly produced by activated Th1 cells) in chickens. In summary, the impact of dietary lipids on gut health may be mediated by direct modification of the fatty acid composition of the mucosal tissues; oxidized fatty acids induce oxidative stress and antimicrobial activities in the intestinal digesta.

Energy is the most expensive nutrient in the poultry diet and is sourced from cereal-based carbohydrates, including corn, wheat, sorghum, oat, rye, and barley, accounting for more than two-thirds of the total energy intake. These cereals contain fibers, including cellulose, NSPs (e.g., arabinoxylans or beta-glucans), resistant starch, oligosaccharides, and non-carbohydrate polysaccharides such as lignin; they can potentially alter the balance of the gut microbiota, intestinal absorptive functions, and immune responses in chickens [192]. It is believed [193] that diets containing high levels of NSPs are known to increase mucin secretion and influence the incidence of NE in broilers via an NSP-mediated increase in digesta viscosity. In addition, an NSP-induced increase in the gut viscosity leads to an increase in the intestinal transit time of the digesta, which induces an increase in bacterial proliferation, especially harmful bacteria, including *E. coli* and *C. perfringens*, and a leaky gut facilitating bacterial translocation from the gut to the blood system [194,195]. Similarly, Yaghobfar and Kalantar [196] found that broilers fed diets containing either wheat or barley have an increased abundance of *E. coli* and *Clostridia* but a lesser abundance of *bifidobacteria* in ileal digesta than the chickens fed a corn–soybean meal-based diet. In addition, chickens fed wheat and barley showed lesser villus heights but increased crypt depths of the small intestine than those fed corn-based diets. Furthermore, the mRNA expression levels of SGLT1 and MUC2 are higher in chickens fed wheat and barley than those fed the corn diet. Paraskeuas and Mountzouris [197] reported that broilers fed a wheat-based diet have higher levels of mucosa-associated *Lactobacillus* and digesta *Bifidobacterium* and lower levels of total bacteria and *Clostridia* clusters I, IV, and XIVa than those fed a corn-based diet. Moreover, wheat-fed chickens have a higher ileal ZO-2 and lower ileal and cecal TLR 2 and sIgA levels than the corn-fed chickens. In contrast to a long belief of the harmful effects of NSPs on gut health, Ghayour-Najafabadi et al. [198] reported that a wheat-based diet increased the relative cecal weight, jejunal villus height, and villus height and crypt depth ratio in broilers than a corn-based diet. They speculated that, compared to corn, dietary wheat might influence the proliferation of *E. coli* and *Lactobacilli,* and the production of acetic and butyric acids, leading to gut health and functional improvement. Recently, it was highlighted that the benefits of NSPs present in cereals on gut health can be attributed to the new cereal cultivars, with low NSP contents, and the next-generation exogenous enzymes targeting NSPs, thus producing prebiotic-like oligosaccharides [10]. The most recognized fiber for gut health is resistant starch. It cannot be digested by host digestive enzymes in the small intestine but can readily be fermented by cecal microflora, producing volatile fatty acids, which stabilize the gut microbiota and improve gut health and function [199].

#### 7.2.3. Impact of Fasting

It is common practice to withdraw the feed for broiler chickens prior to capture, transport, and lairage. This is done to prevent or reduce the incidence of ruptured GI tract and carcass contamination at processing plants by removing the digesta from the GI tract. However, this practice is known to increase the colonization of pathogens due to fasting-induced alterations in the gut morphology and integrity [200], thus compromising gut health. It has been shown that changes in the gut morphology during fasting differ between gut segments [200]. For example, the jejunal villus height and crypt depth increase upon fasting. In contrast, the ileal villus height increases but crypt depth extends and deepens upon fasting, respectively. Thompson and Applegate [200] postulated that an increased villus height during fasting is considered a temporal adaptation to maximize the absorption capacity once the feed is reintroduced into the lumen. It is to be noted that increasing the fasting period decreased the ileal mucus content without affecting the number of goblet cells per villus [200]. Similarly, Yamauchi et al. [201] reported that fasting significantly reduced the villus height of the duodenum and jejunum, but not the ileum, of White Leghorn hens. Thus, fasting compromises the gut health by reducing the overlying mucus layer, which contributes to gut barrier function and protection from pathogens. However, no difference in mucosal thickness of the duodenum, jejunum, and cecum was observed in fasted broiler chickens for 15 h [202].

### 7.3. Environmental Management

#### 7.3.1. Litter Management

Broiler chickens are raised on floor beddings, which are recycled or freshly applied to each flock in different geographical regions. Litter, whether recycled or fresh, is a rich source of microbiota, which will influence the gut microbiome and immune system development of the chicks placed on it upon hatching. Due to the immediate placement of hatched chicks onto the litter, reciprocal interactions between the gut microbiome and litter microbiome have been documented [13]. Wang et al. [203] reported that younger chickens are more susceptible to the litter type than older chickens; the ileal microbiome is affected more than the cecal microbiome. Chickens raised on reused litter have a predominance of butyrate-producing gut bacteria, including *Faecalibacterium prausnitzii.* Similarly, Cressman et al. [204] observed the striking reciprocal effect between the microbiotas present in the litter and those present in the intestine of broilers. Cressman et al. [204] concluded that, compared with used litter, fresh litter impacts the ileal microbiota more than the cecal microbiota; however, the fresh litter-mediated effect on the ileal microbiota decreases with chicken age.

The altered gut microbiota of chickens exposed to fresh or used litter can also influence the local immune indices (e.g., cytokines, immune cell subpopulations, and sIgA). Lee et al. [205] reported that the percentage of T-cell subpopulations (e.g., CD8+ and TCR2+) is higher in chickens raised on used litter than those raised on fresh litter at 14 days. However, after 43 days, chickens raised on used litter showed a lower percentage of CD4+, CD8+, and TCR1+ than those raised on fresh litter. This was the first report demonstrating that the quality of litter (fresh or used) could influence the expression and development of intestinal lymphocyte subpopulations. Lee et al. [206] found that chickens raised on used litter obtained from gangrenous dermatitis-afflicted farms had the lowest levels of cytokine interferon-γ, IL-1β, and IL-10 transcripts than those raised on used or fresh litter for 28 days. The reduced cytokine expression may reflect an overall immune suppression in chickens raised in the used litter obtained from the disease outbreak. Thus, different sources of used litters obtained from different farms have an asynchronous impact on the intestinal immune development of chickens. In contrast to the findings of Lee et al. [206], Shanmugasundaram et al. [207] reported that, compared to broiler chickens raised on fresh litter, those raised on used litter showed an increase in IL-1β and IL-4 transcripts but a decrease in IL-10 transcripts in cecal tonsils. Different cytokine transcript levels observed in the above studies may reflect different genetic backgrounds, ages of chickens, gut commensals, and other environmental factors, such as diet and litter contaminants.

Chen et al. [208] reported that a Chinese native dual-purpose female breed chicken aged 45 weeks, raised in a cage system, exhibited greater microbiome diversity and downregulated most of the Kyoto Encyclopedia of Genes and Genomes pathways in the cecal digesta than did free range-raised chickens. They postulated that the cage-raised chickens may not be exposed to foreign antigens, leading to lower immune stimuli required for innate and acquired immune responses, and tend to have a specific gut microbiome, affecting compromised gut functions and immunity. Thus, the rearing environment (fresh or used litter and cage or floor system) has a huge impact on shaping the gut microbiome compared with cage-reared chickens, which can, in turn, affect the growth and health of chickens. Li et al. [113] also found that floor litter-raised chickens improve the abundance of beneficial bacteria and body weight gain at an early stage of production but lower the indicators of gut health, such as a high abundance of *E. coli,* low volatile fatty acids, and jejunal villus heights and crypt depth ratios, at a later stage of production.

#### 7.3.2. Stocking Density

It is well-documented that the stocking density negatively impacts the gut integrity in the Ross 308 broiler line [209]. For example, increasing the stocking density in the cage rearing system decreases the transepithelial electrical resistance (TER) values as a measure of the intestinal permeability but increases the concentration of lipopolysaccharides (LPS) in the serum of broiler chickens. In addition, the expression of genes encoding ZO-1 and junctional adhesion molecule B (JAM-2) on the jejunal mucosa decreases with the increasing stocking density. In contrast, in a subsequent study by Goo et al. [210], a high vs. low stocking density did not affect the TER values and the expressions of the tight junction-related genes, including ZO-1, occludin, claudin-1, and JAM-2, on the jejunal mucosa. It was postulated that the failure to detect the stocking density-induced decrease in gut integrity in this study could be related to the differences in the stocking density (18.0 vs. 30.4 birds/m^2^) and rearing system (cage vs. floor) compared with the study by Goo et al. [209].

Guardia et al. [211] determined that the stocking density affects the bacterial community in the crop and ceca but not in the ileum of chickens raised on wood shavings at three and six weeks of age. Furthermore, real-time polymerase chain reaction-based quantification of the bacterial groups showed that, compared to a low stocking density, a high stocking density increases the gene copies of the total bacteria, *E. coli*, and *Bacteroides* group in cecal digesta, but not in the crop and ileal digesta, at three weeks of age. However, 16S rDNA gene copies targeted for total bacteria and specific bacteria groups were not affected by the stocking density at six weeks of age. This study indicated that the stocking density affects the bacterial community in younger broiler chickens more than that in older broiler chickens, suggesting a highly volatile propensity of the gut bacterial community of chickens at younger ages. A high stocking density as a contributing factor to NE has been proposed. Tsiouris et al. [212] found that, compared to a high stocking density, a low stocking density increased the number of *C. perfringens* in the cecal digesta of broiler chickens, thus making them susceptible to the development of NE.

#### 7.3.3. Heat Stress

Due to the presence of a feather cover and lack of sweat glands, heat stress can adversely affect the performance, physiology, and the overall health of chickens. Song et al. [213] reported that broiler chickens exposed to heat stress daily during the finisher period lowered their daily weight gain, jejunal villus height, villus height-to-crypt depth ratio, viable counts of *Lactobacillus* in the cecal contents, and TER values in the jejunum, whereas it increased the viable counts of *E. coli* in the cecal contents and the jejunal paracellular permeability. This study highlighted the negative impact of heat stress on the gut integrity and gut microbiome in chickens. As predicted, a Western blot analysis revealed that chickens exposed to heat stress exhibited lower levels of occludin and ZO-1 on the jejunal mucosa than those raised at a thermoneutral temperature [214]. It is known that the tight junction consists of transmembrane protein complexes (e.g., claudins and occludins) and cytosolic proteins (e.g., ZAM-2, ZO-1, ZO-2, and ZO-3). This proves that heat stress induces a significant impact on the dysfunction of tight junction proteins localized onto trans-membranes and in the cytosols. Under acute heat stress lasting five days, heat-stressed chickens had higher mucosal damage of the duodenum and jejunum but not the ileum than the non-heat-stressed chickens at days 1 and 5 following heat exposure [215]. In addition, heat stress decreased both the jejunal villus height and jejunal crypt depth of chickens at days 1 and 5 following heat exposure. Of interest, Santos et al. [215] observed that heat stress exerted different effects on mRNA expressions of genes encoding tight junction proteins in chickens exposed to heat exposure. In general, the expression patterns of genes encoding ZO-1, but not ZO-5, at all segments of the small intestine were greatly upregulated at day 5, but not at day 1, following heat exposure. In the case of claudins, claudin-1, but not claudin-4, was highly upregulated in the jejunal mucosa at day 5 following heat treatment.

Regarding markers for oxidative stress or tissue injury, Santos et al. [215] noted that, compared with the duodenum and ileum, the jejunum is more affected by heat stress. Heat stress particularly upregulated heat-shock protein-70 (HSP70) at days 1 and 5 following heat exposure. However, heat stress downregulated HSP60 at day 1 but upregulated it at day 5 following heat exposure. Similarly, heat stress upregulated the expression patterns of genes encoding for HSP70 and HSP90 in the jejunum and ileum of chickens [216]. However, the HSP70, but not HSP90, protein levels increased in both the jejunum and ileum in chickens exposed to heat stress. Additionally, heat stress increased the mRNA expression of genes encoding for tight junction proteins (claudin-1, claudin-5, and ZO-1) in both the jejunum and ileum [216]. Due to the implication of heat stress-induced gut health, heat stress is considered a predisposing factor for subclinical NE in broiler chickens [217].

#### 7.3.4. Biosecurity Measures

Due to the contamination of poultry origin pathogens (e.g., *Campylobacter* spp.) that cause GI bacterial infection in humans, it is expected that enhanced biosecurity and welfare-related factors at the farm level would prevent the pathogen colonization of poultry. Biosecurity measures at the farm level may include training farm staff, wearing protective clothes, providing house-specific equipment, the collection of dead birds, specifying entry and exit procedures, and disinfection of the facilities. Although biosecurity has been commonly practiced at the farm level, its contribution to gut health in chickens has not been studied. However, the limited literature available suggests that enhanced biosecurity at the farm level might protect chickens against pathogen infection. Georgiev et al. [218] found that practicing enhanced biosecurity at the farm level greatly reduced the colonization of *Campylobacter*, indicating the importance of on-farm measures for pathogen prevention. The impact of farm environment on the poultry microbiome has been recently revealed in two pasture-raised broiler flocks [219]. Although the two farms raised the same chicken breed obtained from the same hatchery and fed the same diets, the physical farm environments influenced the structure and composition of the gut microbiome and the presence of foodborne pathogens (e.g., *Campylobacter* and *Listeria*), highlighting the importance of farm-level ecological dynamics inherent within each farm management system in healthy chicken production. In addition to farm management factors, including litter management, stocking density, and heat stress, further studies are needed to understand whether biosecurity measures at the farm level could influence shaping the gut microbiome, affecting the gut health of chickens.

## 8. Conclusions

A deeper understanding of gut and gut health is vital for the profitable chicken industry facing new global challenges. Gut health is a very broad subject, which involves gut anatomy and physiology, microbiota, immune function, and nutrition. All these different approaches work in an interdependent manner for optimum gut functioning while protecting the gut health in chickens. Although scientists have outlined the factors involved in gut health, defining gut health is difficult due to its dynamic nature.

With the presence of “antibiotic-free” farming conditions, maintaining chicken gut health has become even more challenging. Hence, researchers are studying different factors affecting the chicken gut health and working towards keeping the gut healthy. Host, feed, and environmental factors are being identified as the driving forces that shape the healthy gut conditions in chickens. Moreover, scientists are keen on finding alternative antibiotic growth promoters to enhance performances via promoting gut health.

Nevertheless, a deeper understanding of the gut health and its functional mechanisms will facilitate future advancements of science through sophisticated research tools and techniques to fill the knowledge gaps to maximize the genetic potential of poultry and livestock.
